# Impact of microablative fractional radiofrequency on the vaginal health, microbiota, and cellularity of postmenopausal women

**DOI:** 10.6061/clinics/2020/e1750

**Published:** 2020-07-27

**Authors:** Ayane Cristine Sarmento, Fabíola Sephora Fernandes, Camila Marconi, Paulo César Giraldo, José Eleutério-Júnior, Janaina C. Crispim, Ana Katherine Gonçalves

**Affiliations:** I Programa de Pos-Graduacao em Ciencias da Saude, Universidade Federal do Rio Grande do Norte (UFRN), Natal, RN, BR; II Departamento de Analises Clinicas e Toxicologia, Universidade Federal do Rio Grande do Norte, Natal, RN, BR; III Departamento de Ciencia Biologicas, Universidade Federal do Parana, Parana, PR, BR; IV Departamento de Obstetricia e Ginecologia, Universidade Estadual de Campinas, Campinas, SP, BR; V Departamento de Obstetricia e Ginecologia, Universidade Federal do Ceara, Ceara, CE, BR; VI Departamento de Obstetricia e Ginecologia, Universidade Federal do Rio Grande do Norte (UFRN), Natal, RN, BR

**Keywords:** Menopause, Radiofrequency, Dyspareunia, Orgasm, Therapeutics

## Abstract

**OBJECTIVES:**

We aimed to evaluate the effectiveness of microablative fractional radiofrequency (MAFRF) in the non-hormonal treatment of genitourinary syndrome of menopause.

**METHODS:**

We examined the cases of 55 postmenopausal women before and after treatment with regard to their vaginal health index (VHI), vaginal microbiota, vaginal pH, and cell maturation. Three applications of MAFRF were performed in the vagina/vaginal introitus. During the treatment, six vaginal smears were obtained and stained with the Papanicolaou stain for determining the degree of cell maturation and with Gram stain for classification of vaginal flora, as per the criteria of Spiegel and Amsel. For vaginal pH determination, pH indicator strips were applied against the vaginal wall. Statistical analysis was performed using SPSS for Windows (version 17.0). Data were reported as mean±standard deviation. The differences were analyzed using the statistical method of generalized estimation equations with autoregressive correlation structure “1” and robust standard errors.

**RESULTS:**

The mean age was 59.8±4.2 years, and the mean time of menopause was 15.4±4.5 years. After treatment, there was an increase in the percentage of *Lactobacillus* spp. (*p*<0.001). Consequently, there was a progressive decrease in vaginal pH during the treatment (*p*<0.001). Regarding cell maturation, there was a decrease in the percentage of parabasal cells (*p*=0.001) and an increase in the rate of superficial cells (*p*<0.001). Additionally, there was an improvement in the VHI index. The mean VHI values before and after treatment were 13.2±5.6 and 22.5±3.7, respectively (*p*<0.001).

**CONCLUSION:**

MAFRF treatment is well tolerated and leads to improvement in the vaginal microenvironment.

## INTRODUCTION

Menopause is defined as 12 consecutive months of spontaneous amenorrhea without apparent pathological cause. Genitourinary syndrome of menopause (GSM) is one of most common complaints associated with menopause (1,2).

GSM is defined as a collection of symptoms and signs associated with a decrease in the levels of estrogen and other sex steroids, resulting in changes in the labia majora/minora, clitoris, vestibule/introitus, vagina, urethra, and bladder. Altered epithelial morphology can be observed, *e.g.*, thinning of the vaginal epithelial surface, reduction in fluid secretion and levels of *Lactobacillus,* and consequent increase in vaginal pH (2,3).

Several therapeutic strategies have been proposed for the treatment of GSM. Hormonal (estrogens and androgens) and non-hormonal therapies (lubricants and long-acting vaginal moisturizers) are the most commonly used modalities. It is recognized that vaginal estrogen may improve the symptoms, on the other hand, non-hormonal approaches can be useful in specific cases in which hormonal treatment is not recommended (for instance, breast cancer) (4,5).

Recently, physical methods, such as laser and radiofrequency (RF), in their non-ablative, ablative, and microablative forms have been used to promote neocollagenesis and neoelastogenesis in the vaginal mucosa (6). Currently, some studies have shown good results with laser application to the vaginal mucosa (6-8). Based on the new concept of cutaneous remodeling using optical microscopy patterns of thermal damage with fractional RF on the skin and mucosa, this study aims to evaluate the effects of radiofrequency on the vaginal microenvironment as a possible option in the non-hormonal treatment of GSM.

## MATERIAL AND METHODS

We conducted a prospective study at a gynecological unit of a public university hospital. The first participant was recruited on August 15, 2018 and the last, on October 25, 2019. We included healthy postmenopausal women (55 to 65 years old), who had their last menstrual period (or bilateral oophorectomy) at least 12 months prior to the study, who were sexually active, diagnosed with GSM, had plasma gonadotropin and serum estradiol levels in the postmenopausal range (FSH >40 U/L; estradiol <25 pg/mL), and had negative Papanicolaou (PAP) smear results for cervical cancer precursor cells. Women who had received any form of hormonal (systemic or local) therapy in the last 6 months, used lubricants or vaginal moisturizers in the past month, or had active genital infections or diseases that would interfere with the protocol were excluded.

Microablative fractional radiofrequency (MAFRF) was applied according to the technique described by Kamilos and Borelli (6). For the procedure, the Wavetronic 6000 Touch device was used with the Megapulse HF FRAXX system (Loktal Medical Electronics, São Paulo, Brazil), equipped with an electronic circuit of energy fractionation, connected to a vaginal pen with 64 microneedles, 200 μ in diameter and 1 mm in length, mounted on a Teflon body and divided into an eight-column matrix with eight needles each (6).

In the vestibule and vaginal opening, 10% lidocaine spray was applied 3 minutes prior to the procedure. Three applications were performed in the vagina/vaginal introitus, at intervals of 30 days. Sequential application was performed on the vaginal walls under direct vision. For the post-treatment care, the use of 5% dexpanthenol solution in the vaginal opening was recommended 2-3 times a day for 2 to 5 days, along with abstaining from intercourse for 10 days (6). The procedure was performed in the outpatient clinic by an experienced gynecologist, and a single gynecologist supervised the whole procedure for the entire period of the research.

Six relevant time points were considered for the evaluation of treatment results, based on a previous study (6): baseline (T0), 30 days after first application (T1), 7 days after second application (T2), 30 days after second application (T3), 7 days after last application (T4) and 30 days after the last radiofrequency application (T5). On baseline and days 7 and 30 after each application, vaginal smears were obtained, which were stained according to the standard Gram staining procedure for the classification of vaginal flora as per the criteria of Spiegel and Amsel (9). For vaginal pH determination, the pH indicator strips (MColorpHastTM, Merck, Germany) were applied against the vaginal wall. The material collected from the vaginal sac was distributed on the blade, adequately identified and fixed, and subsequently stained by the Papanicolaou technique for determining the degree of maturation of the vaginal epithelium by the Frost Index.

The vaginal health index (VHI) was determined only at time points T0 and T5. The vaginal health score consists of clinical analysis during specular examination of the five parameters of elasticity, fluid volume, pH, integrity of the epithelium, and humidity, and it is graded from 1 to 5. The sum of the values of the parameters evaluated provides the total vaginal health score. When the total score is less than 15, the vaginal mucosa is considered atrophic (10).

Statistical analysis was performed using Stata 15 (Stata corp., College Station TX, USA). Data presented were reported as mean±standard deviation. The description of changes in variables before (T0) and after treatment (T5) was tested with the paired T-test (Table 1). Changes over time were analyzed with generalized estimation equations with an autoregressive correlation structure of “1” and robust standard errors (Table 2). Statistical significance was set at *p*<0.05.

### Ethics

All study subjects gave informed consent to participate in this research. The study was conducted in accordance with the ethical standards set forth in the Helsinki Declaration (1983), and this protocol was approved by the local Division Ethics Committee (n°81973618.2.0000.5292).

## RESULTS

On evaluating the sociodemographic profile of the 55 women, we found that they were between 55 to 65 years (mean age, 59.8±4.2 years), 39 (71%) were married or with a permanent sexual partner, more than half, 30 (54.5%), received secondary school education and 5 (9%) were from the interior of the state. The time of menopause was 15.4±4.5 years, and 55 (100%) were sexually active.

**Figure 1:**
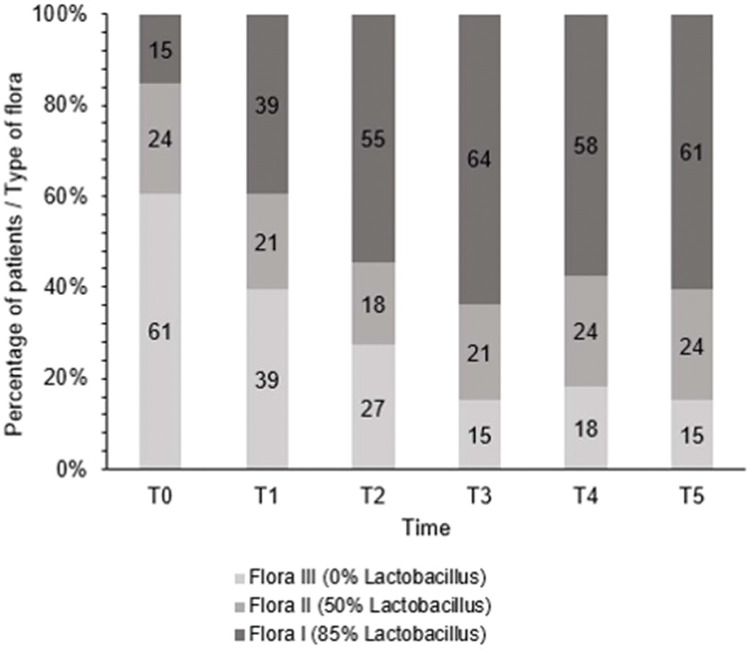
Changes in flora type during treatment.

**Figure 2:**
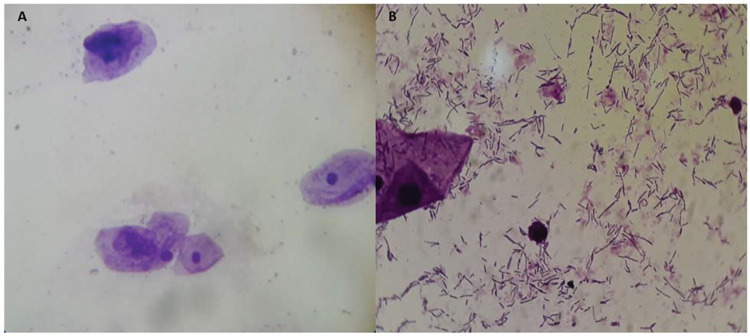
Microscopic observation (Gram staining) of the vaginal microbiota before treatment without *Lactobacillus* (**A**) and after treatment with *Lactobacillus* (**B**).

**Figure 3:**
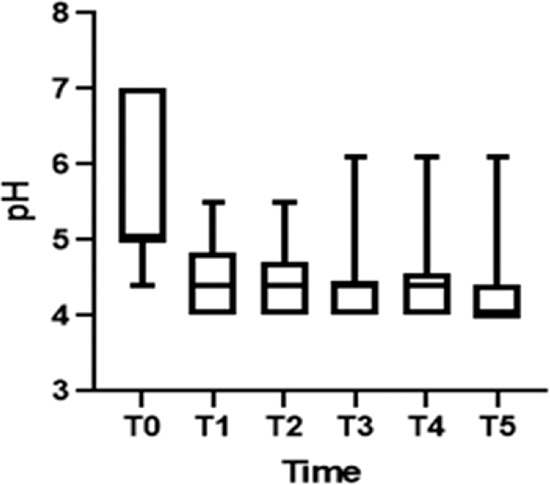
pH levels during the treatment.

After treatment, there was an increase in the level of *Lactobacillus* (Figure 1); this increase in *Lactobacillus* was also observed at the microscopic level (Figure 2). The mean values of *Lactobacillus* at T0, T1, T2, T3, T4, and T5 were 25±33.3, 44.1±38.4, 55.4±36.9, 64.7±31.2, 61.1±32.7, and 63.6±31.0, respectively (*p*<0.001). Consequently, because of the increase in the levels of *Lactobacillus*, there was an apparent change in pH that occurred between the first two assessments. Subsequently, during the follow-up, the pH remained stable in the subsequent evaluations (Figure 3): T0 (5.6±0.9), T1 (4.5±0.5), T2 (4.3±0.4), T3 (4.3±0.4), T4 (4.3±0.4), and T5 (4.3±0.4) (*p*<0.001).

**Figure 4:**
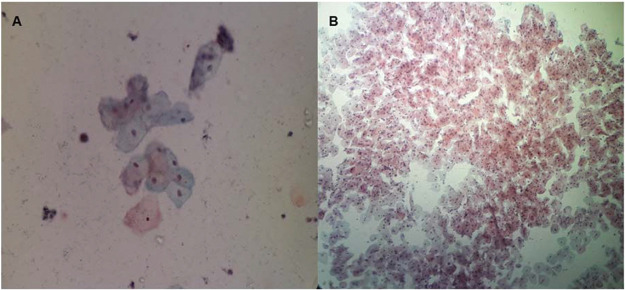
Microscopic observation of the vaginal epithelial cells (Papanicolaou staining) before treatment (**A**) and showing the maturation of the vaginal epithelium after radiofrequency (**B**).

**Figure 5:**
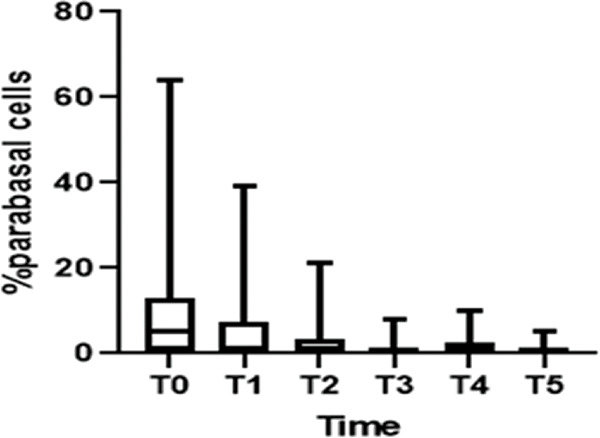
Decrease in parabasal cells.

**Figure 6:**
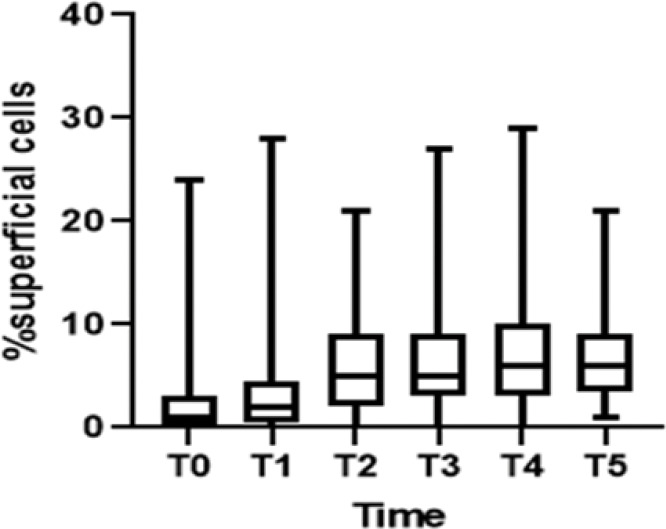
Increase of superficial cells.

Regarding cell maturation, there was a decrease in the percentage of parabasal cells - T0 (9.8±15.7), T1 (4.4±7.6), T2 (2.4±4.5), T3 (1.1±2.1), T4 (1.5±2.5), and T5 (0.8±1.6) (*p*=0.001) - (Figure 4 and 5) and an increase in the percentage of superficial cells - T0 (3.0±5.7), T1 (4.8±7.3), T2 (6.5±5.5), T3 (6.7±6.2), T4 (7.8±7.4), and T5 (7.2±5.0) (*p*<0.001) - (Figure 4 and 6). Additionally, there was also an improvement in the VHI index. The mean values of the VHI baseline before and after treatment were 13.2±5.6 and 22.5±3.7, respectively (*p*<0.001).

The patients recovered quickly, and the aftereffects of microablation disappeared 3-5 days after the application. Concerning the local side effects, just one woman reported burning and redness that lasted 2 to 3 days. None of the patients discontinued the treatment because of the occurrence of adverse events.

## DISCUSSION

Usually, during menopause some symptoms such as hot flushes can be temporary and disappear even without treatment; however, the risk of problems associated with genitourinary syndrome tends to increase with age, and the symptoms may not regress spontaneously, leading to prolonged complaints of urinary/vaginal symptoms and sexual dysfunction (11).

Approximately 50% of menopausal women manifest signs and symptoms of GSM (3). Early diagnosis and active intervention can prevent the appearance of moderate and severe atrophy as well as sequelae. Various therapeutic strategies, hormonal or not, oral or local, have been proposed for improving the vaginal microflora of postmenopausal women. Hormonal therapy (oral or local) has been associated with a healthy vaginal microbiota that is achieved by repopulating the *Lactobacillus* species back to a premenopausal status and by reducing the pH of vaginal fluid (11-13). Additionally, the use of vaginal estrogens reduced the incidence of urinary infections in postmenopausal women (14).

Several clinical studies (5,14) have shown that topical vaginal treatment with estrogen is the gold standard treatment and is associated with effective and rapid improvement of symptoms and complaints. Intravaginal formulations have been developed to reduce systemic exposure to estrogens; previous studies found that topical formulations can also increase estrogen serum levels (15). Thus, caution is advised, particularly for those with a history of estrogen-sensitive cancer, such as endometrial and breast cancers (16). Thus, it is necessary to find non-hormonal options for GSM treatment.

Therefore, a non-hormonal therapeutic option has been proposed for the treatment of urogenital atrophy. Usually, non-hormonal treatments improve vaginal lubrication and dyspareunia after treatment; however, until now, hormonal therapy (estrogen and testosterone) has improved objective parameters, such as pH and vaginal microflora, in most of the women after 12 weeks of treatment. Similar effects can be attributed to treatment with fractional microablative CO_2_ laser (17).

In this study, there was an apparent change in pH that occurred between the first two assessments. Subsequently, during the follow-up, the pH remained stable in the subsequent evaluations, as would be expected if the women had received estrogen therapy. In regard to the increase in the *Lactobacillus* population changing the flora, our results were similar to those of Athanasiou et al. (18), who used laser therapy. They also found that MFCO_2_-laser improved the vaginal flora with a significant reduction in the pH of the vaginal fluid and a significant increase in *Lactobacillus* flora. Additionally, Zerbinati et al. (19) and Salvatore et al. (17) showed that one of the effects of MFCO_2_-laser therapy on the vaginal mucosa was a high degree of epithelial exfoliation, with superficial cells filled with glycogen and shedding in the epithelial surface associated with remodeling of the vaginal connective tissue along with reconstitution of the vaginal mucosa through the activation of regenerative mechanisms, both being expressed in the connective tissue. The formation of new vessels, new papillae, and new collagen in the epithelium could also explain the increase in *Lactobacillus* population and the consequent changes in the vaginal microbiota.

The improvement in the VHI (elasticity, volume of fluid, pH, epithelial integrity, and moisture) was another benefit observed among women treated with radiofrequency. This result is also in agreement with results obtained in previous studies using fractional CO_2_ laser, *e.g.*, Sokol and Karram (20) showed overall mean improvement in VHI scores of 27 women. Salvatore et al. (17,21), also demonstrated that fractional CO_2_ laser treatment was effective after the first application, the VHI improved significantly, with increased improvement after the second and third laser applications.

With regard to the advantages of the use of radiofrequency on the vaginal mucosa when compared to the fractionated CO_2_ laser, considering that the application is carried out under direct vision and with the use of a vaginal speculum, treatment along the vaginal walls is facilitated, preventing overlapping of shots (6). Moreover, the method is easy to learn and affordable. The procedure presented a good tolerance index, with occasional reports of mild discomfort. The patients recovered quickly, and the aftereffects of microablation disappeared 3 to 5 days after the application. No significant adverse effects were observed, and none of the participants had long-term or permanent side effects after the procedure.

The limitations of this study include the relatively small number of participants enrolled, lack of a control arm, and the short follow-up. Given these reasons, the results should be interpreted with caution, but findings of improvement in GSM symptoms were highly significant. The subjects of this current study will be followed-up after 1 year, and those results will be reported in the future. Comparison of existing therapies via randomized controlled trials is currently underway.

Our study did not include a control group, so the results should be considered with caution. However, there was a change in the pH between the first two assessments, with the pH remaining stable in the subsequent evaluations.

## CONCLUSION

Our findings suggest that MAFRF treatment is well tolerated by women and leads to significant improvement in the vaginal microenvironment; therefore, radiofrequency can be used to treat the vaginal symptoms of GSM. The therapy restored the vaginal balance, as would usually be expected with sufficient estrogen levels. The predominance of *Lactobacillus* species and acidic pH of the vaginal fluid achieved after radiofrequency therapy could protect postmenopausal women from vaginal infections, inflammation, and infections of the urogenital tract. However, studies with large samples and extended follow-up periods, focusing on the comparison of microablative fractional radiofrequency with other therapeutic modalities or placebo, are necessary, and one such study is already being carried out by the researchers.

## AUTHOR CONTRIBUTIONS

Sarmento AC, Fernandes FS, and Marconi C were responsible for the study conception and design, data acquisition, analysis and interpretation, manuscript drafting and critical revision. Giraldo PC was responsible for the data acquisition, analysis and interpretation. Eleutério-Júnior J was responsible for the manuscript critical revision. Crispim JC was responsible for the study conception and design, data analysis and interpretation, and manuscript critical revision. Gonçalves AK was responsible for the study conception and design, data acquisition, analysis and interpretation, manuscript drafting and critical revision.

## Figures and Tables

**Table 1:** Description of changes in variables before (T0) and after treatment (T5).

Variable	Difference to the initial value	Standard deviation	95% CI		*p*
pH	-1.24	0.99	-1.59	-0.89	<0.0001
Type of flora	-0.88	0.89	-1.20	-0.56	<0.0001
*Lactobacillus*	38.64	39.57	24.60	52.67	<0.0001
Parabasal cells	-9.00	15.58	-14.52	-3.48	0.0023
Intermediate cells	4.88	15.32	-0.55	10.31	0.077
Superficial cells	4.15	6.32	1.91	6.39	0.0007
VHI	9.33	5.94	7.23	11.44	<0.0001

*Paired T-test.

**Table 2:** Description of changes in variables over time (T0, T1, T2, T3, T4, and T5).

Variable	Variation per unit of time	95% CI		*p*
pH	-0.212	-0.269	-0.155	<0.001
Type of flora	-0.174	-0.235	-0.112	<0.001
*Lactobacillus*	7.637	4.930	10.344	<0.001
Parabasal cells	-1.677	-2.662	-0.692	0.001
Intermediate cells	0.892	0.152	1.935	0.09
Superficial cells	0.837	0.399	1.274	<0.001
VHI	9.333	7.306	11.360	<0.001

*Generalized estimation equations.

## References

[1] [No authors listed] (2013). Management of symptomatic vulvovaginal atrophy: 2013 position statement of The North American Menopause Society. Menopause.

[2] Portman DJ, Gass ML (2014). Genitourinary syndrome of menopause: new terminology for vulvovaginal atrophy from the International Society for the Study of Women’s Sexual Health and The North American Menopause Society. Climacteric.

[3] Wańczyk-Baszak J, Woźniak S, Milejski B, Paszkowski T (2018). Genitourinary syndrome of menopause treatment using lasers and temperature-controlled radiofrequency. Menopauzalny.

[4] Davis SR (2009). Understanding female sexual function. Menopause.

[5] Kingsberg SA, Wysocki S, Magnus L, Krychman ML (2013). Vulvar and vaginal atrophy in postmenopausal women: findings from the REVIVE (Real Women’s Views of Treatment Options for Menopausal Vaginal Changes) survey. J Sex Med.

[6] Kamilos MF, Borelli CL (2017). New therapeutic option in genitourinary syndrome of menopause: pilot study using microablative fractional radiofrequency. Einstein.

[7] Leibaschoff G, Izasa PG, Cardona JL, Miklos JR, Moore RD (2016). Transcutaneous Temperature Controlled Radiofrequency (TTCRF) for the Treatment of Menopausal Vaginal/Genitourinary Symptoms. Surg Technol Int.

[8] Vicariotto F, Raichi M (2016). Technological evolution in the radiofrequency treatment of vaginal laxity and menopausal vulvo-vaginal atrophy and other genitourinary symptoms: first experiences with a novel dynamic quadripolar device. Minerva Ginecol.

[9] Spiegel CA, Amsel LR, Holmes KK (1983). Diagnosis of bacterial vaginosis by direct gram staim of vaginal fluid. J. Clin Microbiol.

[10] Bachmann GA, Nevadunsky NS (2000). Diagnosis and treatment of atrophic vaginitis. Am Farm Physician.

[11] Cano A, Estévez J, Usandizaga R, Gallo JL, Guinot M, Delgado JL (2012). The therapeutic effect of a new ultra low concentration estriol gel formulation (0.005% estriol vaginal gel) on symptoms and signs of postmenopausal vaginal atrophy: results from a pivotal phase III study. Menopause.

[12] Jaisamrarn U, Triratanachat S, Chaikittisilpa S, Grob P, Prasauskas V, Taechakraichana N (2013). Ultra-low-dose estriol and lactobacilli in the local treatment of postmenopausal atrophy. Climacteric.

[13] Caruso S, Cianci S, Amore FF, Ventura B, Bambili E, Spadola S (2016). Quality of life and sexual function of naturally postmenopausal women on ultralow-concentration estriol vaginal gel. Menopause.

[14] Perrotta C, Aznar M, Mejia R, Albert X, Ng CW (2008). Oestrogens for preventing recurrent urinary tract infection in postmenopausal women. Cochrane Database Syst Rev.

[15] Labrie F, Cusan L, Gomez JL, Côté I, Bérubé R, Bélanger P (2009). Effect of one-week treatment with vaginal estrogen preparations on serum estrogen levels in postmenopausal women. Menopause.

[16] Biglia N, Bounous VE, Sgro LG, D'Alonzo M, Pecchio S, Nappi RE (2015). Genitourinary Syndrome of Menopause in Breast Cancer Survivors: Are We Facing New and Safe Hopes?. Clin Breast Cancer.

[17] Salvatore S, França K, Lotti T, Parma M, Palmieri S, Candiani M (2018). Early Regenerative Modifications of Human Postmenopausal Atrophic Vaginal Mucosa Following Fractional CO2 Laser Treatment. Open Access Maced J Med Sci.

[18] Athanasiou S, Pitsouni E, Antonopoulou S, Zacharakis D, Salvatore S, Falagas ME (2016). The effect of microablative fractional CO2 laser on vaginal flora of postmenopausal women. Climacteric.

[19] Zerbinati N, Serati M, Origoni M, Candiani M, Iannitti T, Salvatore S (2015). Microscopic and ultrastructural modifications of postmenopausal atrophic vaginal mucosa after fractional carbon dioxide laser treatment. Lasers Med Sci.

[20] Sokol ER, Karram MM (2016). An assessment of the safety and efficacy of a fractional CO2 laser system for the treatment of vulvovaginal atrophy. Menopause.

[21] Salvatore S, Nappi RE, Zerbinati N, Calligaro A, Ferrero S, Origoni M (2014). A 12-week treatment with fractional CO2 laser for vulvovaginal atrophy: a pilot study. Climacteric.

